# Vertical Columns with Sustainable Green Cover: Meadow Plants in Urban Design

**DOI:** 10.3390/plants12030636

**Published:** 2023-02-01

**Authors:** Violeta Stakelienė, Izolda Pašakinskienė, Kristina Ložienė, Darius Ryliškis, Audrius Skridaila

**Affiliations:** 1 Botanical Garden of Vilnius University, Kairėnų 43, 10239 Vilnius, Lithuania; 2 Life Sciences Centre, Vilnius University, Saulėtekio 7, 10221 Vilnius, Lithuania; 3 Nature Research Centre, Institute of Botany, Žaliųjų Ežerų Str. 47, 08406 Vilnius, Lithuania

**Keywords:** green columns, species dynamics, vegetation classes, environmental factors, Poaceae, *Arabidopsis arenosa*

## Abstract

Unique vertical column structures were constructed for the greening of a structure at the Botanical Garden of Vilnius University, in which a plant cover was formed using the turf rolls of semi-natural meadows that were wrapped on 197 columns, with each column consisting of three equal segments. By evaluating the species composition and the abundance of vegetation in the segments of the columns, we studied how this natural cover changes and what its survival potential is. During the five years of observation, 97 plant species were determined in total. Over time, the initial plant species of fertile soils were mostly replaced by ruderal, nitrophilous, and pioneer plants. Out of the 58 original species, 18 disappeared, while 39 new ones emerged. In the vegetation cover on the north exposition of the building, the original species composition declined faster. The most persistent species were ruderal short-lived *Conyza canadensis*, *Melilotus albus*, and *Urtica dioica,* and long-lived *Elytrigia repens*. As for vegetation classes, the initial plant communities of the *Molinio-Arrhenatheretea elatioris* vegetation class were partially replaced by the plant communities of the *Koelerio-Corynephoretea canescentis* and *Artemisietea vulgaris* classes; however, unformed plant communities finally became prevalent. All directions, including the north, east, south and west, were equally dominated by semi-shade- and semi-light-loving plant species, together with a less abundant representation of light-loving species. Meanwhile, an unexpected establishment of the light-loving annual *Arabidopsis arenosa* was observed on the least illuminated north exposition. Likewise, the perennial *Festuca pratensis*, which is particularly resistant to wintering, emerged and spread on all expositions. The vegetation in the vertical columns was dynamic, and the initial plant species significantly diminished in the five years; however, as new species took place, the columns remained sufficiently covered with a green carpet of plants. This study reveals the benefits of using semi-natural meadow turfs in vertical greening of buildings in the harsh climate of a 5b hardiness zone, which is accompanied by distressing climatic fluctuations during the vegetation season.

## 1. Introduction

With the growth of urbanization, which is one of the most serious issues of the 21st century, the number of urban residents increases rapidly together with the demand for living space [1,2]. As a consequence of urbanization, the request for more green spaces in cities is arising [3]. One of the possibilities of expanding green areas is their integration into building structures. There are two main ways to adapt vegetation in green buildings: green roofs and green walls [4]. Green roofs and walls are sustainable building design elements that have gained attraction and are increasingly used in urban greening [5]. The use of green roofs is a fairly well-established practice worldwide, as flat spaces are easy to use and arrange. However, vertical greening of buildings has a greater impact on the built environment because the surface of a wall is larger than a roof area. It is estimated that the wall area of tall buildings can be as much as 20 times larger than the roof area. In addition, vertical greening improves the thermal properties of buildings [4,6], enhances air quality [1,7,8], increases energy efficiency, reduces noise, improves city aesthetics [3], and reduces stress caused by city life [9,10].

A green vertical system is a self-contained vertical garden attached to the external or internal walls of a building. There are two kinds of structures—“Green Facades” and “Living Walls” [1,4,11,12]. “Green Facades” use climbing plants and special supporting structures. Plants can be planted directly in the ground at the base of the structure or in pots at different levels of the walls [1,4]. Hanging plants and trees are used in the vertical planting of “Living Walls” [4]. This design of greening is particularly suitable for urban buildings, as it allows efficient use of the vertical surface areas [1,12,13]. It is also beneficial for installation in dry heat-affected areas since irrigating water evaporates less than on horizontal surfaces [1,13].

Various types of plants can be used for vertical greening of buildings: vegetables, climbing plants (ivies, roses, and vine grapes), mosses, lichens, alpine plants, and plants of natural flora [1,4,8,14,15]. The implementation of vertical greening systems in urban areas creates habitats for fauna and flora. The choice of plant materials is very important for the planning of greening architecture. Moreover, it is necessary to consider environmental conditions when choosing plant species [2,4]. The use of native plant species creates a landscape design that is harmonious with the nature of the region and is more economical, as native plants are better adapted to the local soil and climate and generally require less irrigation and fertilizer than non-native plants. Therefore, priority should be given to the group of plants that meets environmental conditions with minimal maintenance [16].

In constructing green buildings, obstacles are encountered when choosing plant species to create greater biodiversity together with harmonious aesthetic. In this context, there is a lack of studies that focused on the use of plant species from local flora. The purpose of our experiment was to use the turf rolls from semi-natural meadows in the original vertical columns installed for the greening of a building and to evaluate how such natural cover self-develops and what its sustainability potential is. The purpose of the five-year observation was to determine how the structure of the plant communities develops over time on different expositions (north, south, east, and west) and how the species diversity evolves and adapts while withstanding climatic fluctuations.

## 2. Results

### 2.1. Dynamics of Plant Species

During the five years of observation, 97 plant species were determined in total in the green column structure. Out of the 58 original species, 18 disappeared, while 39 new ones emerged (Table 1).

Perennial plants dominated the plant cover of the vertical structures during the study. From the Poaceae family, the order of abundance was as follows: *E. repens*, *Poa angustifolia*, *Poa pratensis, F. pratensis,* and *Dactylis glomerata* (Table 1). From the Fabaceae family, the order was *Vicia sepium*, *M. albus*, *Medicago falcata*, *Medicago lupulina*, *Trifolium repens*, and *Trifolium pratense*. As could be expected, these species belong to forage plants, and they were installed in the columns with the turf from a semi-natural meadow. The newly occurring species were mostly ruderal plants, namely *Chenopodium album*, *Concoluvulus arvensis, Crepis tectorum, Epilobium parviflorum*, *Euphorbia helioscopia*, *Fallopia convolvulus*, *Impatiens parviflora, Linaria vulgaris, Melilotus officinalis, Moehringia trinervia*, *Silene pratensis*, *Setaria pumila*, *Vicia hirsuta,* and *Viola arvensis* (Table 1).

In each segment of all the expositions, the mean number of species in 2017 was significantly higher than in 2019 and 2021. The number of species in the segments on the north, east, and south expositions did not differ significantly between 2019 and 2021; meanwhile, the number of species in the segments of the west exposition differed significantly in all studied years. In 2017, the segments of the different expositions did not differ significantly by the number of species. The number of species in the northern segments in 2019 was significantly lower compared to the east and west expositions. In 2021, the segments on the north and east expositions differed significantly among themselves by the mean number of species and were characterized by the lowest and highest abundance of species, respectively (Table 2). The mean number of species stopped declining by the last year of observation on the east exposition, but a slight downward tendency remained in the segments on the north, south, and west expositions (Table 2). In all the expositions of the building, the number of species in the segments varied within very wide limits, and the increasing coefficients of variation over the years indicate this enduring tendency (Table 2).

During the five years of study, there was a clear shift in the composition of species in the columns, with some disappearing and others appearing. The most pronounced positive balance in the number of species was recorded in the segments on the east and west expositions, with values of +8 and +9, respectively (Table 3). An evident negative balance between extinct and newly emerging species was observed on the north exposition, with a value of −4. On the south exposition, the species balance was 0 and +1, suggesting that as many species disappeared in the segments as approximately the same number appeared (Table 3).

### 2.2. Dynamics of Plant Communities

In the first year of the study (2017), more than two-thirds of the segments were covered by the plant communities that belonged to the *Molinio-Arrhenatheretea elatioris* R. Tx. 1937 phytocoenological vegetation class, i.e., the primary plant communities that were installed as the turf rolls into the vertical columns from a semi-natural meadow (Table 4 and Table 5). In the rest of the segments, unformed plant communities prevailed, except for one segment on the east exposition where the plant community belonged to the *Nardetea strictae* Rivas Goday et Borja Carbonell vegetation class. In 2019 (the third year of the study), more than ½ of all the segments remained predominately covered by the plant communities of the *Molinio-Arrhenatheretea elatioris* vegetation class. However, later on, the frequency of the plant communities belonging to the *Molinio-Arrhenatheretea elatioris* vegetation class rapidly decreased, and in 2021 (the fifth year of the study), the plant communities of this vegetation class came close to extinction (only identified in 1.7% of the segments). The assessment of the last year (2021) showed that unformed plant communities began to predominate in the vertical green columns, as these unformed plant communities were found in >60% of the segments (Table 4). In addition, at this time, the plant communities belonging to the *Koelerio-Corynephoretea canescentis* Klika et Novak 1941 and the *Artemisietea vulgaris* Lohm., Prsg et R.Tx. 1950 vegetation classes made up a considerable part of the green cover, occurring at 23.2% and 13.0% of the segments, respectively.

**Figure 1:**
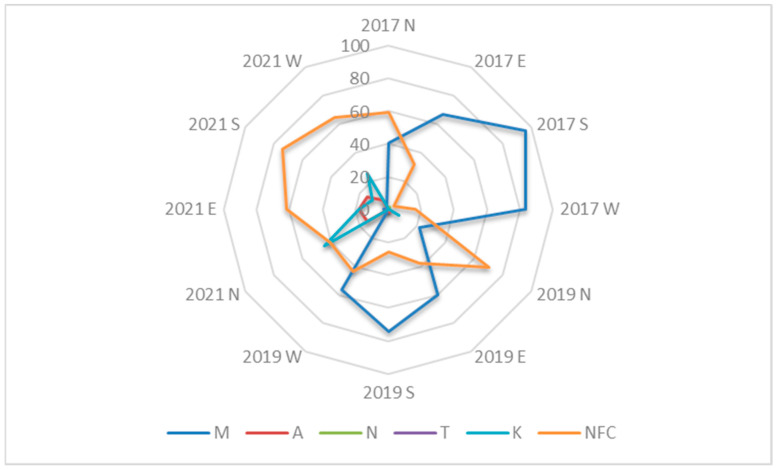
Dynamics of plant vegetation classes in the segments of the vertical green columns in relation to the expositions of the building (north, east, west, and south) (M—Cl. *Molinio-Arrhenatheretea elatioris*; A—Cl. *Artemisietea vulgaris*; N—Cl. *Nardetea strictae*; T—Cl. *Trifolio-Geranietea sanguinei*; K—Cl. *Koelerio-Corynephoretea canescentis*; and NFC—unformed communities).

Differences in vegetation development were observed depending on the column exposition (Figure 1). It was found that in 2017 and 2019, the south, east, and west expositions were predominated by the original plant communities of the *Molinio-Arrhenatheretea elatioris* vegetation class, but in 2021, almost all of them were replaced by unformed plant communities (Figure 1). A different situation was observed in the segments on the north exposition, where, already in the first year, the number of unformed plant communities exceeded the number of plant communities assigned to the *Molinio-Arrhenatheretea elatioris* vegetation class, and in 2019, this difference clearly shifted in favor of the former (Figure 1). In the last year of observation, the plant communities belonging to the *Molinio-Arrhenatheretea elatioris* vegetation class in the north side almost disappeared, while the communities of the *Koelerio-Corynephoretea canescentis* vegetation class and unformed plant communities were present to a similar extent in the segments (Figure 1). The plant communities of the *Artemisietea vulgaris* vegetation class were not recorded in the first year of the study. However, this type appeared in 2019 in one segment located on the east exposition. By 2021, communities of the *Artemisietea vulgaris* vegetation class were formed on all the expositions at a rate of 13% of the total segments (Figure 1, Table 4). Overall, the five years of observations showed that, in the vertical green columns, the initial plant communities belonging to the *Molinio-Arrhenatheretea elatioris* vegetation class declined and were largely replaced by unformed plant communities.

### 2.3. Light Requirement and Species Distribution

**Figure 2:**
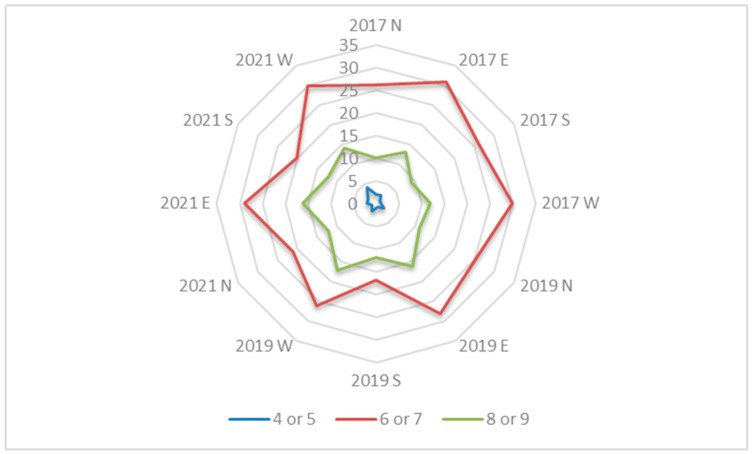
Species assignment according to their light preference using Ellenberg’s indicator values and their distribution in the segments of the vertical green columns in different expositions (north, east, west, and south) in the study years 2017–2022. Ellenberg’s indicator values: 3—shade plants, mostly less than 5% illumination; 4—between 3 and 5; 5—semi-shade plants, rarely in full light, but generally with more than 10% relative illumination; 6—between 5 and 7; 7—plants generally in well-lit places, but also occurring in partial shade, and not found in less than 30% relative illumination; 8—light-loving plants found in relative illumination in summer no less than 40%; and 9—plants in full light, found mostly in full sun, and with illumination in summer no less than 50%.

Based on the assumption that, in our green column construction, the greatest influence on the development of the plant cover should be sunlight radiation exposure, we divided the species according to their requirement for light using Ellenberg’s indicator values and assessed their distribution in the different expositions (Table 1, Figure 2). The majority of plant species belonged to the group of semi-shade-loving plants (61.2%), half as many were semi-light-loving plants (31.8%), and there were relatively few species of light-loving plants (7.0%) (Table 1). After evaluating the dynamics of the species, we found that semi-shade- and semi-light-loving species prevailed in all the expositions during the five-year period (Figure 2). During the last years, a slight increase in the number of light-loving plants was observed on account of a reduction in semi-shade- and semi-light-loving plants (Figure 2). Even though the overall balance of the number of species in the entire column system was positive (Table 3), the trend of species number decline was evident on the south and north exposition segments (Figure 2).

**Figure 3:**
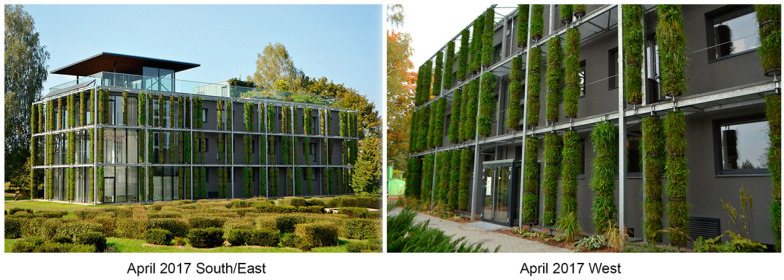
The green columns during the first year’s spring season on the south/east and west expositions, April 2017.

**Figure 4:**
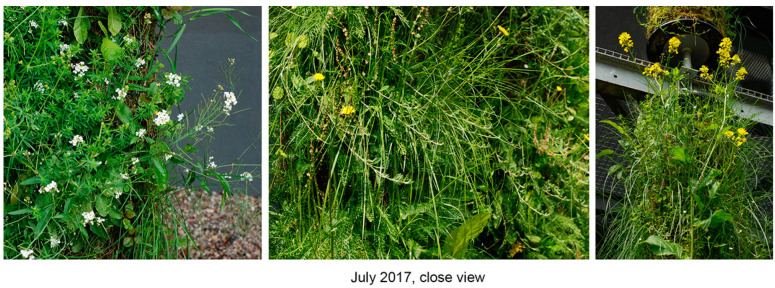
An example of plant species representation in the green columns, July 2017.

**Figure 5:**
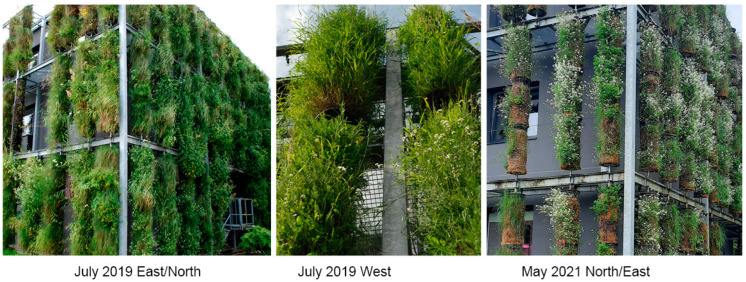
The green columns during the third year’s summer season on the east/north and west expositions, July 2019; *A. arenosa*’s dominant representation in the fifth year’s spring season on the east/north exposition, May 2021.

**Figure 6:**
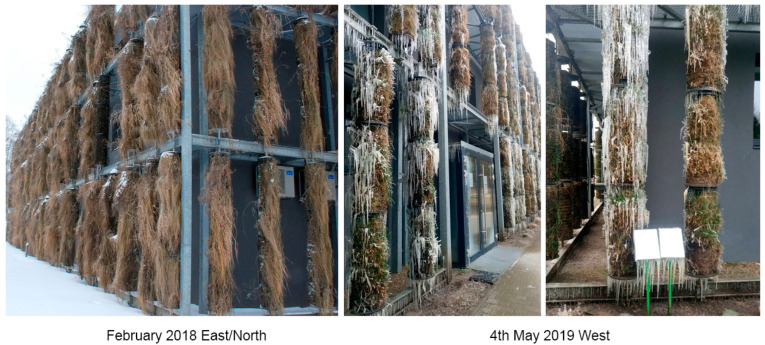
Winter view of the green columns on the east/west exposition, February 2018; the damaging effect of a sudden frost in the green columns in the early spring season on the west exposition, 4 May 2019.

We found that, over the course of five years, the initial composition of plant species from local semi-natural meadows declined; however, as new species actively took over, the columns remained sufficiently covered with green plant layers (Figure 3, Figure 4 and Figure 5). Our five-year assessment showed that the turfs installed in the vertical columns for a building’s greening allowed the plants to survive seasonal fluctuations characterized by sudden frosts in spring and frequent heat waves in summer (Figure 6, Table 6).

## 3. Discussion

We installed a unique vertical column structure for the greening of a building at the Botanical Garden of Vilnius University. This green construction is unique because we used turf rolls cut from semi-natural meadows at the local site and wrapped them on 197 columns, each comprised of three equal segments. Our study allowed us to observe how the vegetation cover of semi-natural meadows changes in four different expositions of the building (north, south, east, and west). Ninety-seven plant species were recorded in the plant cover of the column segments during the five years of observation.

The columns we installed are unique not only due to the construction but also due to the meadow turf used. There is very little literature on a similar topic. Our study could be most comparable to the work of Stollberg and co-authors [18]. They used seed mixtures of natural meadow species in the construction of green walls and came up with a lot of useful technical experience. However, low seed germination (horizontal pre-growing stage) required a lot of re-sowing and resulted in uncovered gaps in the green layer, which was a disadvantage in terms of labor and time. Another group of researchers, Campiotti and coauthors [19], used a monospecies of *Parthenocissus quinquefolia* in building vertical greenery and analyzed the beneficial effect on reducing the heat temperatures in a Mediterranean region. Despite the fact that we did not measure such physical parameters, we assume that our green column structure significantly shaded the building and, thus, had an impact on its protection from the extreme heat in summer.

Regardless of the exposition, the initially dominant plant communities of the *Molinio-Arrhenatherethea elatioris* vegetation class were gradually replaced by unformed plant communities. In addition, the plant communities of the *Koelerio-Corynephoretea canescentis*, *Artemisietea vulgaris*, *Nardetea strictae,* and *Trifolio-Geranietea sanguinei* vegetation classes appeared in some segments of the green columns. In Central Europe, plant communities of the *Molinio-Arrhenatherethea elatioris* vegetation class are the most common [20]. Therefore, it is not surprising that this class of plant communities was initially detected as being the most abundant in the segments of the vertical structures formed by the turf of semi-natural meadows. Compared to others, plant communities belonging to the *Molinio-Arrhenatherethea elatioris* vegetation class are the most productive, comprising natural and semi-natural mesophyte communities that thrive in sufficiently moist and fertile soils. Meanwhile, communities of other vegetation classes occur in soils of different fertility levels characterized by different soil acidity, salinity, and moisture. *Koelerio-Corynephoretea canescentis* plant communities are common on seacoasts and continental sands with gravelly soils; *Artemisietea vulgaris* plant communities are established in areas with no humus soil layer; *Nardetea strictae* plant communities are formed in infertile, more acidic soils; and *Trifolio-Geranietea sanguinei* plant communities are found in warm and dry woodlands, forest sites, and meadow slopes [21,22,23,24].

In our study, the plant species in the green columns were disturbed when the meadow cover pieces were laid on these vertical structures. Thus, the plants were exposed and affected by a particular anthropogenic activity. Anthropogenic changes in the environment are known to promote the decline of species diversity [25]. As regards the species composition, *Galium aparine*, *Stellaria media*, *E. repens*, *Achillea millefolium*, *P. angustifolia*, *P. pratensis*, *Rumex acetosa*, *T. pretense*, and *T. repens* were found to be dominant in the vertical columns in the first year. Later, *E. repens* and *A. millefolium* remained dominant, even though their coverage in the segments decreased. The majority of the newly appearing species were ruderal plants, including *Ch. album*, *Concoluvulus arvensis*, *C. tectorum*, *E. parviflorum*, *E. helioscopia*, *F. convolvulus*, *I. parviflora*, *L. vulgaris*, *M. officinalis*, *M. trinervia*, *S. pratensis*, *S. pumila*, *V. hirsuta,* and *V. arvensis*, i.e., species that establish themselves in damaged or disturbed environments in open areas. It is likely that the seeds of these ruderal plants entered the columns with the composted soil that was used to fill the segments of the structure or from the seeds present in the initial turf layer, since ruderal plants are known to be the accompanying components in the vegetation of meadows [26].

*M. albus*, *U. dioica*, and *C. canadensis* were highly persistent during the experiment. Poaceae species also played an important role in green cover formation and development. This is likely due to their intensive colonization capability and persistence in ecosystems under different environments [27]. During the five-year study, the abundance of *P. pratensis* ceased, but *F. pratensis* spread prevalently; *D. glomerata* disappeared, while the coverage and abundance of *E. repens* increased. This could be the consequence of the original fast cover-forming species diminishing and leaving a free space for other species to establish themselves. T. Sasaki and W. K. Lauenroth [15] observed a similar trend in the plant communities of a shortgrass steppe. Their study showed that the loss of the dominant, rapidly cover-forming species might reduce the competition and alter the hierarchy of the dominant species in plant communities. It was shown that the removal of the dominant species *Bouteloua gracilis* from a long-term experimental site increased species diversity, the number of rare species, and the relative abundance of dominant species [15]. In another study conducted by Stollberg and co-authors [18], it was observed that species diversity increased after the disappearance of *Stachys palustris*, *G. album*, and *Galium palustre*. This corresponds to our observation that the loss of the initial dominant species in the vertical columns has led to the establishment and dominance of new species. Our results also correspond with the studies by Mårtensson and co-authors [28], and, thus, we can say that the vertical structure had a negative impact on the establishment of dominant species.

No threat in terms of the establishment of invasive plant species in the vertical columns was identified. Invasive *Lupinus polyphyllus* was recorded on all sides of the building in the first year of the study, and later, a single case of invasive *I. parviflora* species was observed. However, overall, it can be stated that the unnatural position of the meadow turf in the vertical structures prevented the establishment of invasive plants.

In our green column system, five plant species, *Cerastium holosteoides*, *Mentha arvensis*, *Urtica urens*, *Sonchus oleraceus*, and *Tanacetum vulgare*, were the most sensitive to the change from natural to vertical vegetation arrangement in the columns, as they disappeared already after the first two years. These species are found in nature close to cultivated fields, near homesteads, and on roadsides [29,30,31,32]. Perhaps, these species did not establish themselves in the altered habitat due to some climatic factors. This could possibly account for sudden frosts early in the season and/or severe heat during the summer. Temperature fluctuations and their extreme values are classified as the most important limiting factors for plant growth [28]. Generally, high temperatures have a negative impact on grassland species diversity [33]. In addition, the scale and frequency of extreme weather events are increasing, and so are climatic fluctuations, which affect changes in species diversity [34]. In addition, one of the negative factors could have been the absence of snow cover, and so the plants were more exposed to the cold in winter. Another reason could be the intra- and interspecific competition of plants for the availability of nutrients [35], as no fertilizers were applied in our system.

The obtained data were also analyzed based on the assumption that sunlight exposure was clearly a variable depending on the column exposition. The south exposition was the most illuminated. The east and west ones also had enough light, but on the north side, the plants grew in shade or semi-shade. Our observations showed that the different duration and intensities of light were likely to be the significant factors in the rearrangement of the species composition of the cover. Notably, on the north exposition, a faster loss of initial plant species and a greater establishment and dominance of nitrophilic and pioneer plants were observed than on other sides of the building. This shows that the most intensive changes in vegetation took place in the least illuminated vertical columns. As a result, this could be the reason why *M. falcata* and *V. sepium* disappeared on the north exposition, and the coverage of *M. lupulina* decreased. *V. sepium* frequency and coverage also decreased on the east and west expositions. Meanwhile, *Ajuga reptans,* which naturally grows in deciduous and mixed forests, woodlands, and forest slopes [32], decreased in number and coverage on all the expositions except on the north side.

Notably, we observed exceptional cases when some plant species successfully adapted and began to dominate in conditions that are not naturally characteristic of them. Light-loving *A. arenosa* plants (indicator value nine according to Ellenberg) appeared and established themselves in the segments on the north side, the least illuminated exposition. This is likely because of *A. arenosa*’s extraordinary ability in colonizing new places [36]. In contrast, in the columns of other expositions, the frequency and coverage of *A. arenosa* were lower, possibly due to the fast coverage effect of other rapidly growing species. M. Stollberg and coauthors [18] observed a similar trend, where the extinction of dominant species allowed other species to become established. Besides that, their study showed that light is not always the determining factor for plant dominance [18]. Therefore, in our study, when *A. arenosa* experienced less competition, shade was no longer a critical factor for this species to grow and complete its vegetation cycle. Meanwhile, the spread of *F. pratensis* in the columns can be explained by the high sustainability of this perennial grass in the 5b hardiness zone [37], which includes the south-eastern part of Lithuania where the Botanical Garden of Vilnius University is located [38].

## 4. Methods and Materials

### 4.1. Installation of Vertical Columns

**Figure 7:**
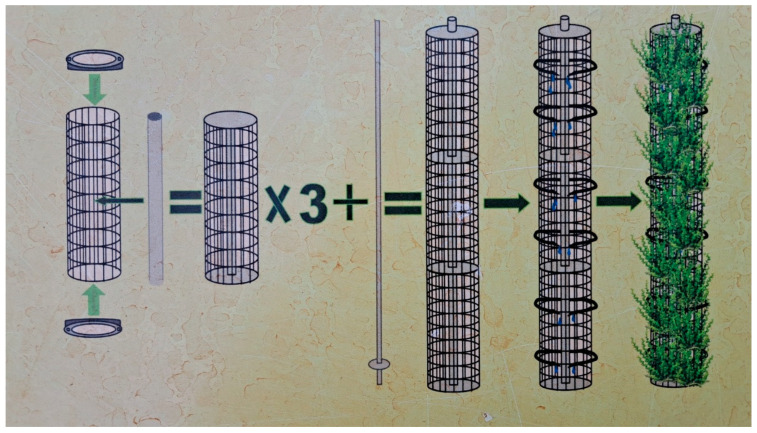
The vertical column construction scheme.

The vertical structures were installed in August–September 2016 for the greening of the Administrative Laboratory building at the Botanical Garden of Vilnius University (Lithuania, Vilnius; N54.7362067, E25.4034823). Thirty-four columns were installed on the north, 75 on the east, 21 on the south, and 67 on the west exposition of the building (Figure 3, Table 5). Each column consisted of three segments (0.30 m in diameter and 0.90 m high) connected by a metal frame axis passing through a plastic hollow tube in the centre of the segments (Figure 7). The segment constructions were filled with composted soil, and turf rolls cut from the semi-natural meadows at the Botanical Garden were wrapped around the axis to make a plant cover. Fertile compost soil from the Botanical Garden was used. An agrotextile film was inserted between the compost soil and the meadow turf to retain moisture. The meadow turf was reinforced from the outside with a wire mesh. Each segment was covered with plastic covers at the top and bottom. An irrigation system was installed inside the columns. A plastic pipe was attached along the entire upper line of the column’s construction, from which four capillaries were inserted into each segment. Water was supplied through the capillaries. The total surface area of all the columns was 585 m^2^, i.e., 1.5 times large than 362 m^2^ wall area of the building.

### 4.2. Maintenance of the Columns

Plants grown in the vertical columns were not cut during their growing season. Dead plant biomass was removed annually after winter in April. The columns were watered every day. Each of the three levels of the segmental columns had separate water supply sections (12 sections in total). Water was supplied periodically, for 5 min every 3 h daily, during the vegetation period of the plants. In the late season, when the plants stopped growing, irrigation was turned off. The plants were not fertilized since the columns were installed. The plant cover on the columns was not cut during the vegetation season, and the columns were not additionally filled with compost soil.

### 4.3. Plant Species Composition and Vegetation Assessment

The study was conducted over a five-year period from 2017 to 2021. The plant species composition, the projection coverage, and the abundance of each species in the vertical structures were assessed in 2017 (the first year of the experiment), 2019 (the third year) and 2021 (the fifth year), once a year in the same period. The columns were randomly selected for assessment; they were the same throughout the years of the study. Nine columns (27 segments) were evaluated on the north and south expositions, 21 columns (63 segments) were evaluated on the east exposition, and 20 columns (60 segments) were evaluated on the west (Table 5) exposition. In total, 177 segments in 59 columns were monitored. The plant species composition was assessed in each segment separately on the area of the entire segment. The Braun-Blanquet scale [39] was used for the evaluation of the coverage abundance of different species. The plant communities were distinguished according to the vegetation classification systems proposed by J. Balevičienė [21], J. Balevičienė et al. [22], and W. Matuszkiewicz [23].

### 4.4. Analysis of Meteorological Data and Ecological Parameters

**Figure 8:**
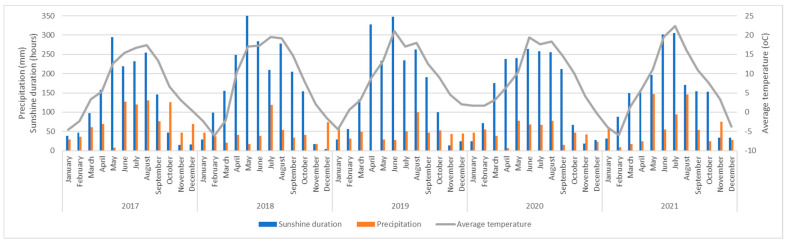
Dynamics of climatic environmental factors in Vilnius over the study years 2017–2021.

The meteorological parameters were obtained from the nearby Vilnius Automatic Meteorological Station of the Lithuanian Meteorological Service, whose coordinates are N54.625992, E25.107064. Lithuania is located in the mid-latitude climate zone and belongs to the southwestern subregion of the Atlantic continental forest area. On average, plant vegetation starts in the first decade of April and ends by the first decade of October. The monthly physical environmental parameters, the mean air temperature, and the duration of sunshine and precipitation for the study years 2017–2021 are presented in Figure 8.

No particular severe winter conditions were specified; the winters in 2017–2021 were mild, and minus temperatures did not exceed the limits defined for a USDA 5b hardiness zone. The winter of 2019–2020 was exceptionally mild, with the lowest temperatures of −5–−8 °C occasionally recorded, and the mean was above zero, at 1.8°C in comparison to −2.9 °C for the long-term mean.

Heat waves were common in summer during the study years (Table 6). In 2019, the months of July and June were extremely hot: the mean temperature reached 22.4 °C in July and 19.5 °C in June (hottest since 1961). The incidences of stressful meteorological conditions, such as frosts in spring and summer heat waves, which can affect plant vegetation, are listed in Table 6.

### 4.5. Statistical Analysis and Calculations

One-way analysis of variance (ANOVA) was used to assess the differences in the number of species in the segments of the vertical columns between different expositions and different years. The statistical analyses were carried out using STATISTICA^®^ 7 and MS Excel Software. The diagrams of meteorological and ecological parameters and plant community dynamics were drawn using MS Excel Software.

## 5. Conclusions

In summary, we make the following conclusions: (i) the vegetation cover of a semi-natural meadow turf changes significantly when it is used in artificial vertical columns, and the number of initial plant species is reduced by about a third; (ii) however, due to the establishment of a high number new species, the columns remain sufficiently covered by green plant layers for up to five years. The original plant species of the fertile soils were mostly replaced by ruderal, nitrophilous, and pioneer plants. Out of the 58 original species, 18 disappeared, while 39 new ones emerged. In the vegetation cover of the north exposition, the original species composition dwindled most rapidly, and the most active replacement with newly emerging species occurred. The five-year observations showed that the original meadow plant communities belonging to the *Molinio-Arrhenatheretea elatioris* vegetation class were partially replaced by the plant communities of the *Koelerio-Corynephoretea canescentis* and *Artemisietea vulgaris* vegetation classes; however, unformed plant communities finally became prevalent. The columns on all the sides of the building were equally dominated by semi-shade- and semi-light-loving plant species, together with less abundant light-loving species. The most persistent species were ruderal short-lived *C. canadensis*, *M. albus*, and *U. dioica,* and long-lived *E. repens*. In some instances, the loss of the dominant, rapidly cover-forming species led to the apparent establishment of new species. The most active newly spreading species were the annual *A. arenosa* and the perennial *F. pratensis*. Our study shows that the turfs of local semi-natural meadows are suitable for the installation of vertical columns for greening of buildings in the harsh climate of a USDA 5b hardiness zone, which is accompanied by sudden climatic fluctuations, including frosts in the early season and heat waves in the summer.

## Figures and Tables

**Table 1:** The presence (sp and Roman numerals), abundance, and coverage (Arabic numerals and symbols in the degree) of plant species in the vertical column segments by year and by the exposition of the columns.

Year	2017				2019				2021				
Side of the Building	N	E	S	W	N	E	S	W	N	E	S	W	
Coverage (%):													The light indicator values according to Ellenberg [17]
herbs	88	95	95	92	77	95	91	88	73	90	85	83	
bryophytes	34	54	42	48	27	49	40	41	18	42	35	26	
*Achillea millefolium*	IV^+−3^ *	IV^+−4^	V^+−4^	IV^+−4^	III^+−2^	IV^+−3^	IV^+−3^	III^+−3^	I^+−1^	II^+−3^	II^+−2^	III^+−2^	8–9
*Agrimonia eupatoria*	sp^+^	I^+^	I^+−1^	sp^+^	–	–	–	sp^+^	–	–	–	–	6–7
*Ajuga reptans*	sp^+^	sp^+^	sp^+−1^	sp^+−1^	sp^+−1^	sp^+−2^	sp^+^	sp^+^	I^+−2^	sp^+^	sp^+^	sp^+^	6–7
*Alchemilla subcrenata*	–	sp^+^	–	–	–	sp^+^	–	–	–	–	–	–	6–7
*Anthemis tinctoria*	sp^1^	sp^+^	–	–	–	sp^+^	–	–	–	sp^+^	–	–	8–9
*Arabidopsis arenosa*	–	–	–	–	V^+−4^	III^+−3^	III^+−1^	II^+−2^	V^+−5^	IV^+−4^	IV^+−3^	III^+−4^	8–9
*Arenaria serpyllifolia*	sp^1^	–	–	II^+−3^	–	–	–	I^+−2^	–	sp^+^	–	sp^+^	n
*Artemisia campestris*	sp^1^	sp^+−1^	sp^+−2^	sp^+^	sp^+^	sp^1−2^	sp^+−2^	sp^+^	–	sp^2^	sp^+^	sp^+−1^	8–9
*Artemisia vulgaris*	sp^+^	–	sp^+^	sp^+^	sp^+^	sp^+^	sp^+^	sp^+^	sp^+^	sp^+^	sp^+^	sp^+−1^	6–7
*Barbarea vulgaris*	–	sp^+^	–	–	–	–	sp^+−2^	–	–	–	–	–	8–9
*Betula pendula*	–	–	–	–	sp^+^	–	–	–	–	–	–	–	6–7
*Bidens tripartita*	–	–	–	sp^+^	–	–	–	sp^+^	–	–	–	–	8–9
*Capsella bursa-pastoris*	sp^+−2^	sp^+−2^	sp^+−2^	sp^+−1^	–	–	–	sp^+^	–	sp^+^	sp^1−2^	sp^+^	6–7
*Carex hirta*	–	–	–	–	sp^+^	–	–	–	sp^1^	sp^+−1^	–	–	6–7
*Carum carvi*	I^+−1^	sp^+−1^	sp^+^	sp^+^	–	sp^+^	–	sp^+^	–	sp^+^	–	–	8–9
*Centaurea scabiosa*	–	sp^+^	–	sp^+^	–	sp^+^	–	–	–	–	–	–	6–7
*Cerastium arvense*	sp^+^	sp^+^	–	–	–	–	–	–	–	–	sp^+^	sp^+^	8–9
*Cerastium holosteoides*	sp^+^	sp^+^	–	–	–	–	–	–	–	–	–	–	6–7
*Chelidonium majus*	–	–	–	–	sp^1^	–	–	–	–	–	–	–	6–7
*Chenopodium album*	–	–	–	–	–	–	–	–	sp^+^	sp^+−1^	sp^+^	sp^+^	n
*Chenopodium glaucum*	–	–	–	–	–	–	–	–	–	–	sp^+^	–	8–9
*Convolvulus arvensis*	–	–	–	–	–	–	–	–	–	–	–	sp^+^	6–7
*Conyza canadensis*	–	–	–	sp^+^	–	–	–	sp^+^	sp^+^	sp^+^	sp^+^	sp^+^	8–9
*Crepis tectorum*	–	–	–	–	–	–	–	–	sp^+^	–	–	–	8–9
*Dactylis glomerata*	sp^+^	sp^+−2^	I^+−2^	I^+−2^	sp^+−2^	sp^+−2^	sp^+−1^	I^+−2^	–	–	–	–	6–7
*Elytrigia repens*	III^+−3^	II^+−3^	II^+−2^	II^+−3^	IV^+−4^	IV^+−3^	IV^+−3^	III^+−3^	IV^+−4^	III^+−5^	IV^+−5^	III^+−4^	6–7
*Epilobium parviflorum*	–	–	–	–	–	–	–	–	II^+−1^	II^+−2^	sp^+^	sp^+−1^	6–7
*Equisetum arvense*	–	–	–	–	–	sp^+^	–	–	–	sp^+−2^	–	sp^+−1^	6–7
*Erigeron acris*	–	–	–	–	–	–	–	–	sp^+^	sp^+^	–	–	8–9
*Euphorbia helioscopia*	–	–	–	–	–	sp^+−2^	–	–	–	sp^+^	–	–	6–7
*Euphrasia stricta*	–	–	–	–	–	sp^+−1^	sp^+^	sp^+^	–	sp^+^	–	sp^+−2^	n
*Fallopia convolvulus*	–	–	–	–	–	–	–	–	sp^+^	–	–	sp^+^	6–7
*Festuca pratensis*	–	–	–	–	–	–	–	–	I^+−2^	I^+−2^	II^+−2^	II^+−2^	6–7
*Fragaria vesca*	sp^+^	sp^+−2^	I^+−2^	sp^+−1^	sp^+^	I^+−2^	I^+−2^	sp^+−2^	sp^+^	sp^+^	sp^+^	sp^+^	6–7
*Galium album*	–	–	–	–	–	–	–	–	sp^+−1^	I^+−2^	sp^+^	sp^+^	6–7
*Galium aparine*	I^+−3^	III^+−3^	II^+−2^	III^+−2^	sp^+−1^	II^+−3^	II^+−2^	III^+−3^	I^+^	II^+−2^	I^+−3^	II^+−2^	6–7
*Geranium palustre*	–	–	–	–	–	sp^+^	–	sp^+^	sp^+^	II^+−2^	I^+−1^	I^+−2^	8–9
*Geranium pratense*	–	–	–	–	sp^+−2^	sp^+^	sp^+−2^	sp^+^	–	–	–	–	8–9
*Geranium sibiricum*	–	–	–	–	–	sp^+^	sp^+^	sp^+^	–	–	–	–	n
*Geum urbanum*	sp^+−2^	sp^+^	–	–	–	–	–	–	–	–	–	–	4–5
*Glechoma hederacea*	II^+−2^	I^+−2^	II^+−2^	II^+−1^	sp^+^	sp^+^	sp^+^	sp^+^	I^+−3^	sp^+^	I^+^	I^+−2^	6–7
*Heracleum sibiricum*	–	–	–	–	–	–	–	–	–	sp^+^	–	–	n
*Hypericum perforatum*	sp^+^	sp^+−1^	sp^+^	sp^+^	–	sp^+^	–	–	sp^+^	sp^+^	–	sp^+^	6–7
*Impatiens parviflora*	–	–	–	–	–	–	–	–	–	–	–	sp^+^	4–5
*Knautia arvensis*	sp^+^	I^+−1^	sp^+−1^	I^+−1^	sp^+^	sp^+−2^	sp^+^	sp^+−2^	–	sp^+−1^	sp^+^	–	6–7
*Lapsana communis*	–	–	–	–	sp^+^	–	–	sp^+^	–	–	–	sp^+−1^	4–5
*Leontodon autumnalis*	–	–	–	–	sp^+^	sp^+^	–	–	sp^+−1^	sp^+^	sp^+−1^	sp^+^	6–7
*Leucanthemum vulgare*	sp^+^	sp^+^	sp^+^	sp^+−2^	–	sp^+^	–	sp^+^	–	–	–	–	6–7
*Linaria vulgaris*	–	–	–	–	–	–	–	sp^+^	–	sp^1−2^	–	sp^+^	8–9
*Lotus corniculatus*	sp^+^	sp^+−1^	II^+−2^	sp^+^	–	sp^+^	–	sp^+^	–	–	–	–	6–7
*Lupinus polyphyllus*	I^+−1^	sp^+−2^	sp^+−2^	sp^+−1^	–	–	–	sp^+^	–	–	–	–	6–7
*Medicago falcata*	sp^+^	II^+−3^	sp^+−3^	I^+−2^	sp^+^	II^+−3^	I^+−4^	I^+−3^	–	II^+−4^	sp^+−3^	I^+−4^	8–9
*Medicago lupulina*	sp^2^	sp^+−2^	sp^+−2^	sp^+−2^	sp^+−1^	I^+−3^	sp^+−3^	I^+−2^	sp^+^	I^+−2^	I^+−2^	I^+−2^	6–7
*Melilotus albus*	–	sp^+−2^	sp^+^	sp^+^	sp^+^	sp^+^	I^+−2^	I^+−2^	sp^+−1^	II^+−4^	II^+−5^	II^+−5^	8–9
*Melilotus officinalis*	–	–	–	–	–	–	–	–	–	sp^2^	–	–	8–9
*Mentha arvensis*	–	sp^+^	–	–	–	–	–	–	–	–	–	–	6–7
*Moehringia trinervia*	–	–	–	–	–	–	–	–	–	sp^+−2^	sp^+−2^	sp^+^	4–5
*Phleum pratense*	–	–	–	–	sp^+^	–	–	–	–	–	–	–	6–7
*Pilosella officinarum*	I^+−1^	II^+−3^	II^+−2^	II^+−3^	–	sp^+^	–	sp^+−1^	–	–	–	sp^+^	6–7
*Pimpinella saxifraga*	–	–	–	–	sp^+^	I^+−1^	II^+−1^	I^+−2^	sp^+^	sp^+−2^	sp^+^	sp^+−2^	6–7
*Plantago lanceolata*	sp^+^	sp^+^	–	sp^+^	III^+−3^	sp^+−2^	sp^+−3^	II^+−3^	sp^+^	sp^+−1^	–	sp^+−3^	6–7
*Plantago major*	–	–	–	–	–	–	sp^+^	–	–	–	sp^+^	–	n
*Plantago media*	sp^+^	sp^+^	sp^+^	sp^+^	sp^+^	sp^+−1^	sp^+−2^	sp^+^	–	sp^+^	sp^+−2^	sp^+^	6–7
*Poa angustifolia*	IV^+−4^	V^+−5^	V^+−4^	V^+−4^	II^+−3^	IV^+−4^	IV^+−3^	IV^+−3^	sp^+−1^	II^+−3^	II^+−3^	II^+−3^	6–7
*Poa pratensis*	II^+−3^	II^+−4^	II^+−4^	III^+−3^	II^+−2^	II^+−3^	II^+−3^	III^+−2^	I^+−2^	I^+−2^	I^+−2^	I^+−1^	6–7
*Potentilla anserina*	–	–	–	–	sp^+−2^	–	–	–	sp^+^	–	–	–	6–7
*Potentilla argentea*	II^+−1^	sp^+^	I^+^	sp^+^	I^+−1^	sp^+−1^	sp^+−1^	sp^+−1^	sp^+^	–	–	sp^+−1^	8–9
*Primula veris*	II^+−2^	sp^+−1^	sp^+−2^	sp^+−2^	sp^+^	sp^+−2^	–	sp^+−3^	sp^+^	sp^+^	sp^+^	sp^+−2^	6–7
*Prunella vulgaris*	sp^+^	–	–	sp^+^	–	–	–	sp^+^	–	–	–	–	6–7
*Ranunculus acris*	–	–	–	sp^+^	sp^+^	I^+−1^	sp^+^	sp^+^	sp^+^	sp^+^	–	sp^+^	n
*Ranunculus auricomus*	–	–	–	–	–	–	–	–	–	–	sp^+−2^	–	4–5
*Ranunculus repens*	–	sp^+−1^	sp^+^	sp^+^	–	–	–	sp^+^	–	–	–	–	6–7
*Rumex acetosa*	II^+−2^	I^+−3^	III^+−2^	I^+−2^	II^+−2^	I^+−2^	II^+−2^	I^+−2^	sp^+^	I^+−2^	sp^+−1^	I^+−2^	8–9
*Silene pratensis*	–	–	–	–	–	–	–	–	–	–	–	sp^+^	8–9
*Silene vulgaris*	–	sp^+−2^	–	–	sp^+^	sp^+^	sp^+^	sp^+−2^	sp^+^	I^+−2^	–	sp^+^	8–9
*Sedum acre*	–	–	–	–	–	sp^+−2^	–	–	–	sp^+−2^	–	–	8–9
*Senecio jacobaea*	–	–	–	sp^+^	–	–	–	sp^+^	–	–	–	–	8–9
*Setaria pumila*	–	–	–	–	–	–	–	–	–	–	–	sp^+^	6–7
*Setaria viridis*	–	sp^+^	–	–	–	–	–	–	sp^+^	sp^+^	–	–	6–7
*Solidago virgaurea*	–	–	–	–	sp^+−1^	sp^+^	sp^+^	sp^+^	sp^+^	sp^+−1^	sp^+^	sp^+^	n
*Sonchus oleraceus*	–	sp^+^	sp^+^	sp^+^	–	–	–	–	–	–	–	–	6–7
*Stellaria holostea*	sp^+^	II^+−3^	II^+−3^	I^+−3^	I^+−4^	I^+−1^	II^+−2^	II^+−3^	sp^+^	I^+−2^	I^+−2^	sp^+−3^	4–5
*Stellaria media*	III^+−4^	III^+−4^	III^+−3^	II^+−2^	–	sp^+^	sp^+−3^	–	sp^+^	sp^+^	–	sp^+^	n
*Stellaria graminea*	–	–	–	–	–	–	–	–	–	sp^+−2^	–	sp^+−2^	6–7
*Tanacetum vulgare*	–	–	–	sp^1^	–	–	–	–	–	–	–	–	6–7
*Taraxacum officinale*	sp^+^	sp^+^	I^+^	sp^+^	sp^+^	sp^+−1^	sp^+−2^	sp^+^	sp^+^	sp^+^	sp^+^	sp^+^	6–7
*Thymus pulegioides*	sp^+^	I^+−4^	sp^+−2^	I^+−2^	sp^+^	II^+−3^	sp^+−2^	II^+−3^	sp^+−1^	II^+−2^	I^+−2^	I^+−2^	8–9
*Trifolium pratense*	II^+−3^	II^+−3^	III^+−3^	III^+−2^	sp^+^	sp^+^	sp^+^	I^+−1^	–	sp^+−1^	sp^+^	sp^+^	6–7
*Trifolium repens*	I^+−1^	II^+−2^	II^+−2^	I^+−2^	sp^+^	I^+−2^	sp^+^	I^+−3^	sp^+^	sp^+^	–	sp^+−1^	8–9
*Tussilago farfara*	–	–	–	–	–	–	–	–	–	–	sp^+^	–	8–9
*Urtica dioica*	sp^+−1^	sp^+^	sp^+^	sp^+−1^	I^+−2^	sp^+^	sp^+^	sp^+^	I^+−3^	sp^+^	I^+−2^	sp^+−2^	n
*Urtica urens*	–	sp^+^	–	–	–	–	–	–	–	–	–	–	6–7
*Verbascum nigrum*	sp^+^	I^+−1^	sp^+^	I^+−1^	sp^+^	sp^+^	–	sp^+−1^	–	sp^+^	–	sp^+−1^	6–7
*Veronica chamaedrys*	II^+−2^	sp^+−1^	sp^+^	I^+−2^	sp^+−1^	sp^+^	sp^+^	sp^+−2^	–	sp^+^	–	sp^1^	6–7
*Vicia hirsuta*	–	–	–	–	–	–	–	–	–	sp^+^	–	–	6–7
*Vicia sepium*	sp^+−1^	II^+−3^	II^+−2^	II^+−3^	I^+−2^	II^+−3^	II^+−4^	II^+−3^	–	I^+−2^	II^+−4^	II^+−2^	n
*Viola arvensis*	–	–	–	–	–	–	–	–	–	–	–	sp^+^	6–7

* 1–5—the scale of the minimum and maximum projection coverage of the plant species; n—no Ellenberg’s indicator value is assigned.

**Table 2:** Descriptive characteristics of the number of plant species in the investigated segments of the vertical columns, as well as a one-way analysis of variance (ANOVA) examining years and building expositions according to the number of plant species in the segments (CV—coefficient of variation (%); SD—standard deviation; df—degree of freedom; F—Fisher criterion; *p*—statistical significance; *—significant differences between years (*p* < 0.05); **—significant differences between expositions of the building (*p* < 0.05). Letters denote statistically significant (*p* < 0.05) differences: capital letters—between the years in each exposition, lower letters—between expositions each year).

Exposition		Statistical Parameter		Year			One-Way ANOVA Testing Years (df = 2)	
				2017	2019	2021	F	*p*
North		Mean ± SD		12.1 ± 3.2 ^A,a^	8.6 ± 2.9 ^B,a^	7.4 ± 3.9 ^B,ac^	14.02	6 × 10^−6^ *
		Min–Max		5–21	3–15	2–19		
		CV (%)		26	34	52		
East		Mean ± SD		12.5 ± 3.0 ^A,a^	10.5 ± 2.9 ^B,bc^	10.5 ± 3.0 ^B,b^	10.12	7 × 10^−5^ *
		Min–Max		5–20	5–21	3–18		
		CV (%)		24	28	29		
South		Mean ± SD		13.4 ± 2.6 ^A,a^	10.4 ± 2.3 ^B,ac^	9.6 ± 2.6 ^B,cb^	17.29	10^−6^ *
		Min–Max		10–21	6–16	5–15		
		CV (%)		19	22	27		
West		Mean ± SD		13.7 ± 3.3 ^A,a^	11.5 ± 2.9 ^B,bc^	9.1 ± 3.1 ^C,cb^	32.65	9 × 10^−13^ *
		Min–Max		7–21	7–18	4–16		
		CV (%)		24	25	34		
One-way ANOVA testing building expositions (df = 3)			F	2.50	6.67	6.23		
			*p*	0.06	0.0003 **	0.0005 **		

**Table 3:** The shift in the number of plant species in the segments of the green columns according to the exposition of the columns.

Exposition	2019			2021			Total Species Balance for Two Years
	Extinct Species	Emerging Species	Species Balance	Extinct Species	Emerging Species	Species Balance	
North	14	12	–2	14	12	–2	–4
East	12	13	+1	9	16	+7	+8
South	11	11	0	13	14	+1	+1
West	5	10	+5	13	17	+4	+9

**Table 4:** Distribution of plant vegetation classes in the segments of the vertical green columns in different years.

Vegetation Classes	Percentage of Investigated Segments in the Columns		
	2017	2019	2021
*Molinio-Arrhenatheretea elatioris*	72.9	55.9	1.7
*Artemisietea vulgaris*	0.0	0.6	13.0
*Nardetea strictae*	0.6	0.0	0.0
*Koelerio-Corynephoretea canescentis*	0.0	1.1	23.1
*Trifolio-Geranietea sanguinei*	0.0	0.0	0.6
Unformed plant communities	26.5	42.4	61.6

**Table 5:** Structural description of the green columns.

Exposition	Installed		Evaluated	
	Number of Columns	Number of Segments	Number of Columns	Number of Segments
North	34	102	9	27
East	75	225	21	63
South	21	63	9	27
West	67	201	20	60

**Table 6:** Overview of severe meteorological incidents in the study years 2017–2021.

Year	Severe Meteorological Incidents	
	Sudden Frosts in Spring/Low Temperatures	Summer Heat Waves
2017	Severe frosts during the period of early vegetation on 9–14 May and 17 May, and the temperature was down to −1.7…−4.0 °C.	None
2018	None	The first heat wave was on 30 May, when the highest air temperature reached 30.3 °C. The second heat wave occurred on 20 July–4 August, when the temperature reached 31 °C. The third heat wave occurred on August 8–10, when the temperature exceeded 30 °C.
2019	Frost which lasted for 6 days during the period of early vegetation on 4–9 May, and the temperature was down to −2.1…−3.8 °C.	In June, there were two heat waves on the 4–7 and 11–14, when the temperature rose up to 34.2 °C.
2020	Frost during early vegetation was the most damaging and lasted for several days on 8–12 May, and the temperature was down to −1.8…−3.5 °C.	Three heat waves were recorded on 11 June, 20 July, and 16 August, when the temperature reached 28.8–30.3 °C.
2021	Active plant vegetation was significantly delayed (about 10 days) due to a long period of low temperatures in April–May (≤10 °C)	A long heat wave during the active growing season on 18–25 June, with the highest temperature reaching 33.1 °C on 23 June.

## Data Availability

All the data are included in the main text.
